# Three Dimensional Printing and Biomaterials in the Repairment of Bone Defects; Hydroxyapatite PLA Filaments

**DOI:** 10.3906/sag-1901-184

**Published:** 2019-06-18

**Authors:** İsmail KAYA, Meryem Cansu ŞAHİN, İlker Deniz CİNGÖZ, Nevin AYDIN, Murat ATAR, Ceren KIZMAZOĞLU, Salih KAVUNCU, Hasan Emre AYDIN

**Affiliations:** 1 Department of Neurosurgery, Faculty of Medicine, Kütahya University of Health Sciences, Kütahya Turkey; 2 Department of Radiation Oncology, Evliya Çelebi Training and Research Hospital, Kütahya Turkey; 3 Department of Neurosurgery, Alsancak Nevvar Salih İşgören Public Hospital, İzmir Turkey; 4 Department of Radiology, Faculty of Medicine, Eskişehir Osmangazi University, Eskişehir Turkey; 5 Department of Neurosurgery, İstanbul Sultan Abdülhamid Han Training and Research Hospital, İstanbul Turkey; 6 Department of Neurosurgery, Faculty of Medicine, Dokuz Eylül University, İzmir Turkey; 7 Department of Plastic Reconstructive and Aesthetic Surgery, Faculty of Medicine, Afyon University of Health Sciences, Afyonkarahisar Turkey; 8 Department of Neurosurgery, Faculty of Medicine, Kütahya University of Health Sciences, Kütahya Turkey

**Keywords:** Hydroxyapatite, PLA, filaments, bone tissue engineering, three dimensional printing

## Abstract

**Background/aim:**

Application fields of bone tissue engineering studies continue to expand. New biocompatible materials aimed to improve bone repairment and regeneration of implants are being discovered everyday by scientists, engineers, and surgeons. Our objective in this study is to combine polylactic acid which is a polymer with hydroxyapatite in the repairment of bone defects considering the increased need by medical application fields.

**Materials and methods:**

After 750 g of PLA with a diameter of 2.85 mm was granulated into minimum particles, these particles were homogenously mixed with hydroxyapatite prepared in laboratory environment. Using this mixture, HA-PLA filament with a diameter of 2.85 mm was prepared in the extrusion device in Kütahya Medical Sciences University Innovative Technology Laboratory. The temperature was 250 °C and the gearmotor speed was 9 rpm during extrusion. X-ray diffraction (XRD) analysis was made for crystal phase analyses of the produced hydroxyapatite powder, to determine the produced main phase and examine whether a minor phase occurred. Vickers microhardness test was applied on both samples to measure the endurance levels of the samples prepared with HA-PLA filament. A loading force of 10 kg was applied on the samples for 10 s.

**Results:**

Hydroxyapatite peaks in XRD spectrum of the sample presented in figures are concordant with Joint Committee on Powder Diffraction Standards, JCPDS - File Card No. 01-075-9526 and no significant minor phase was observed. For both samples, hardness value was observed to increase between 3 and 5 mm.

**Conclusion:**

Surfacing hydroxyapatite on metallic materials is possible. By similar logic, to increase durability with low cost, characteristics of biomaterials can be improved with combinations such as hydroxyapatite PLA. Thus, we found that while these materials have usage limitations due to present disadvantages when used alone, it is possible to increase their efficiency and availability through different combinations.

## 1. Introduction

Application fields of bone tissue engineering studies continue to enlarge. New biocompatible materials aimed to improve bone repairment and regeneration of implants are being discovered everyday by scientists, engineers, and surgeons. 

Biocompatible, safe, and efficient biomaterials with a gradually increasing importance and field of application today are materials used to fulfill and support the functions of organs and living tissues in human body [1]. 

Hydroxyapatite, collagen, hyaluronic acid, poly fumarates, poly caprolactone, polylactic acid (PLA), polyglycolic acid (PGA), and PLA and PGA copolymers are some of the biomaterials used in medical field. Characteristics of these materials such as durability and compatibility were increased by combinations. In addition to implant applications, biomaterials are now used for bone tissue regeneration. Repair work of biomaterials on hard tissues such as bone began with ‘bone bonding’ defined by Williams et al. in 1992 and has advanced quickly since then [2].  

Our objective in this study is to combine polylactic acid which is a polymer with hydroxyapatite in the repairment of bone defects considering the increased need by medical application fields. 

## 2. Materials and methods

Materials and method used in this study are covered in detail under the headings “Hydroxyapatite Powder Synthesis through Hydrothermal Synthesis Method”, “Preparation of HA-PLA Filament” and “Characterization”.

### 2.1. Hydroxyapatite powder synthesis through hydrothermal synthesis method

**Figure 1:**
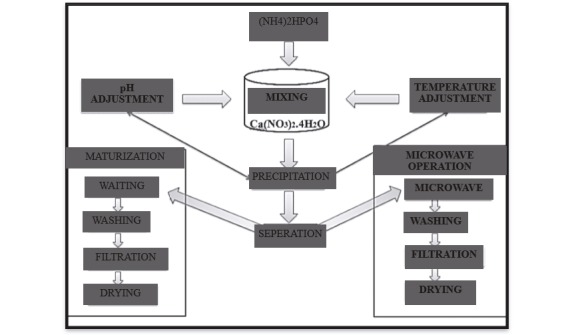
Hydroxyapatite acquisition through hydrothermal synthesis method.

To form 1 mol of Stock solution, 11.807 g of calcium nitrate tetra hydrate (Ca(NO3)2.4H2O) and 6.603 g of diammonium hydrogen phosphate ((NH4)2HPO4) were dissolved in 50 mL of distilled water. Stock solution was diluted with distilled water to acquire 0.10 M solutions and then suspensions with 1.67 Ca/P molar ratios were acquired by adding drops of 10.0 mL of 0.10 M diamonium hydrogen phosphate to 16.7 mL of 0.10 M calcium nitrate tetrahydrate. The pH value of the acquired suspensions was measured as 5.0. The prepared solutions were transferred to hydrothermal reactors with a volume of 125 mL. Hydrothermal reactors were left to normal cooling after controlled heating at 200 °C in an incubator for 24–72 h. The produced powder was centrifuged through distilled water and ultrasonic bath method and this operation was repeated for six times until the pH value reached 7. The acquired hydroxyapatite powders were left to dry at 50 °C in an oven for 4 h (Figure 1). 

### 2.2. Preparation of HA-PLA filament

**Figure 2:**
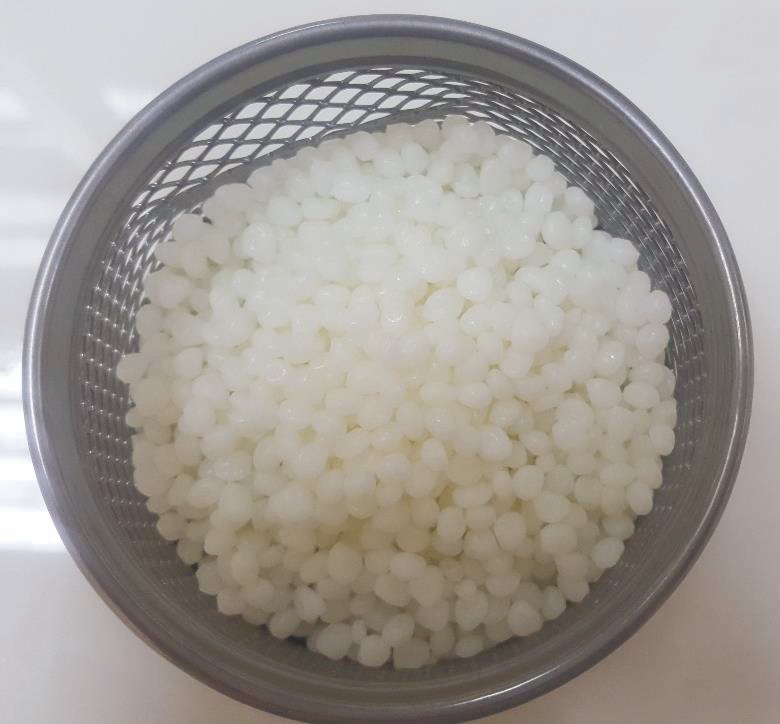
Granulated PLA.

After 750 g of PLA with a diameter of 2.85 mm was granulated into minimum particles, these particles were homogenously mixed with hydroxyapatite prepared in laboratory environment. Granulated PLA was purchased from Sigma-Aldrich with GF45989881-1EA product code. The granules with the size of 3 mm were provided in packages of 100 g (Figure 2). 

**Figure 3:**
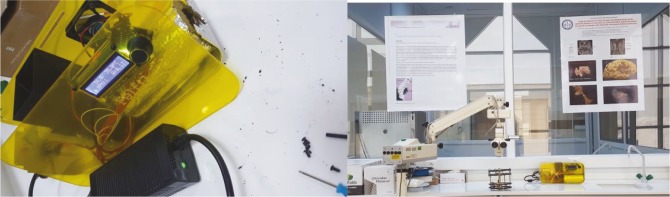
Extrusion device and HA-PLA filament preparation.

Using this mixture, HA-PLA filament with a diameter of 2.85 mm was prepared in the extrusion device in Kütahya Medical Sciences University Innovative Technology Laboratory (Figure 3). The temperature was 250 °C and the gearmotor speed was 9 rpm during extrusion.

Using HA-PLA filament and pure PLA filament prepared for analysis, two circular samples with a diameter of 5 cm and height of 2 cm were prepared in Ultimaker 3 Extended brand three dimensional printer. In order to obtain an effective result, the size of 5 cm diameter and 2 cm height was chosen in production phase. Many implants used in medical area like spinal cage, spinal screw, and maxilla plates have similar sizes. Thus, our product which was generated equivalent to different medical implant sizes will give us guiding mechanical test results for our future studies. During printing, bed temperature was adjusted as 100 °C and nozzle temperature as 280 °C for HA-PLA filament and bed temperature was adjusted as 70 °C and nozzle temperature as 240 °C for pure PLA filament.

### 2.3. Characterization

X-ray diffraction (XRD) analysis was made for crystal phase analyses of the produced hydroxyapatite powder, to determine the produced main phase and examine whether a minor phase occurred. XRD data were acquired in 10°–70° 2θ range with 0.02° step range and 5°/min scanning speed. 

Vickers microhardness test was applied on both samples to measure the endurance amount of the samples prepared with HA-PLA filament. A loading force of 10 kg was applied on the samples for 10 s. The test was performed at room temperature. Measurement was taken from four different points randomly on the surfaces of the samples. 

In biomaterial studies in the literature, 100 gr force and 15-s time protocols were applied for Vicker’s hardness test of pure PLA. However, the results we got from the literature review show that mechanical strength of hydroxyapatite PLA mixture has increased. Besides, the HA-PLA filament produced will be used for implant production in the future studies. Considering human anatomy, the current implant materials are supposed to be stable to avoid possible damages because of body weight or environmental factors. Thus, we have preferred 10 kg in our study. However, we are working on increasing this weight in our progressing animal tests and continuing our in vivo and in vitro studies. 

## 3. Results

### 3.1. XRD findings for hydroxyapatite produced through hydrothermal synthesis method

**Figure 4:**
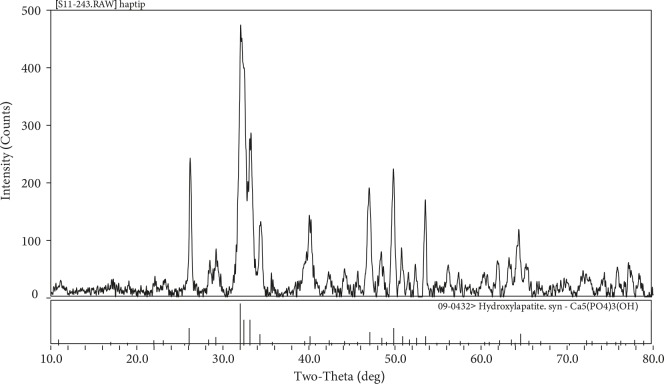
XRD spectrums of hydroxyapatite produced through hydrothermal synthesis method.

XRD spectrums of hydroxyapatite produced through hydrothermal synthesis method are presented in Figure 4. Hydroxyapatite peaks in XRD spectrum of the sample presented in Figure 4 are concordant with Joint Committee on Powder Diffraction Standards, JCPDS - File Card No. 01-075-9526 and a significant minor phase was not observed.

### 3.2. Vickers microhardness test findings of samples produced with both filaments

**Figure 5:**
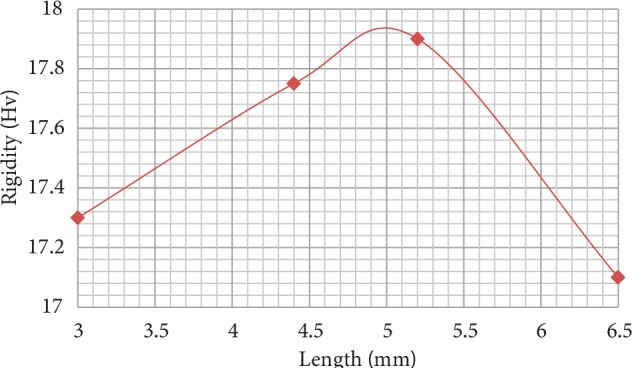
Results of the microhardness test for the sample produced with pure PLA filament.

**Figure 6:**
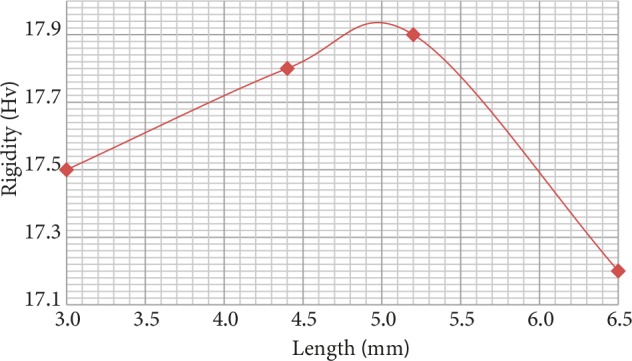
Results of the microhardness test for the sample produced with HA-PLA filament.

Results of Vickers microhardness test performed on samples produced with two different filaments are presented in Figures 5 and 6. Hardness value of the sample produced with pure PLA filament was measured as nearly 17 Hv. Hardness value of the sample produced with filament acquired from hydroxyapatite and PLA mixture was measured nearly 17.5 Hv. For both samples, hardness value was observed to increase between 3 and 5 mm.

The hydroxyapatite nano size powder produced with hydrothermal synthesis is in the shape of a particle. In order to implement hydroxyapatite mechanical tests for this size, different processes are needed and powder hydroxyapatite needs to get pellet size. Considering the existing material amount and process time, this period makes the experiment model difficult. The most important reason why we preferred HA-PLA mixture is the search for a guiding material to produce implant in our future studies. As it is known, using hydroxyapatite alone and in shape of powder is chemically and mechanically impossible for implant production. Our study’s main purpose is making an alternative way for studies conducted just using PLA in the literature by testing hydroxyapatite and PLA mixture tolerance. Thus, not just hydroxyapatite mechanical tests will provide positive data in our study. 

## 4. Discussion

Autografts, allografts, and xenografts have been used for a long time for reconstruction of bone tissue. Although there were studies in 1990s showing that autogenous cortical grafts are durable and efficient, necessity of an additional surgical cut and donor site morbidity has limited the usage area. [3,4]. 

Metal implants such as steel and titanium started to be used to repair bone defects, with advanced technology. Hydroxyapatite coverings are used today instead of metal grafts which have no benefit to bone regeneration. Hydroxyapatite, which is a bone structure, is more efficient in bone fusion and because of this advantage it began to be utilized in defect repair [5]. 

Biocompatibility is the most important characteristic for biomaterials and can be examined both for structure and surface compatibility. Surface compatibility is the physical, chemical, and biological compatibility of a biomaterial to body tissues. Structural compatibility is the optimum compatibility of the material to the mechanical behavior of body tissues [6]. Developing a biocompatible biomaterial imitating natural bone cell is one of the most important part to be considered in bone tissue engineering. Materials that can be used instead of many bones such as bioactive glass, bioactive ceramics, natural or synthetic polymers and their composites have been investigated in detail in the last twenty years [6]. Hydroxyapatite (Ca10(PO4)6(OH)2), tricalcium phosphate and bioactive glasses are clinically interesting bioactive inorganic materials. Among these materials, hydroxyapatite was used commonly in orthopedic implants due to its resemblance to natural bone [7]. However, clinical applications of hydroxyapatite are limited due to its fragility.

As biopolymers; collagen, hyaluronic acid, polyfumarates, polycaprolactone, polylactic acid (PLA), polyglycolic acid (PGA), PLA and PGA copolymers are alternative candidates for tissue engineering. PLA is preferred in bone tissue engineering due to its biocompatibility. PLA-based composites promise a high potential hence more and more studies are conducted in biomedical field, especially bone tissue engineering. Biodegradable synthetic polymers such as Polylactic Acid are the most commonly used materials in three dimensional tissue scaffolds in terms of biodegradability and thermal plasticity [8]. When used alone, it is difficult for polylactic acid or hydroxyapatite to meet all criteria required for a specific application area [9]. Thus, composite materials are required to improve mechanical, biocompatibility, and biodegradability characteristics of available biomaterials. Polylactic acid and hydroxyapatite are saturated Poly-α-hydroxy esters [10]. Polylactic acid which is one of these esters is sometimes called polylactide and has three forms as the racemic mixture of poly (D-lactic acid) (PDLA), poly (Lactic acid) (PLLA) and D, L-PLA (PDLLA). When compared to other aliphatic polyesters, PLA has excellent characteristics such as machinability, high mechanical strength, high module, biocompatibility, biodegradability, low toxicity, transparency, and availability for use in three dimensional printing [11,12]. Screws, pins, and plates can be easily manufactured to be used in orthopedic applications.

Controlling of cellular responses was focused on for the formation of new tissues through the biomimetic approach for improving biomaterials for bone tissue regeneration. For this purpose, osteoconductive and also osteogenesis stimulating osteoinductive materials which physically have a structure resembling extracellular matrix are utilized. At this point, as it resembles the mineral phase in human bone structure in chemical and biological terms, hydroxyapatite (HA), Ca10(PO4)6(OH)2 is the most preferred implant material in orthopedics and dentistry. Having a hexagonal rhombic cage structure, HA plays an active role in the provision of the mineral balance of the bone and preservation of mechanical strength. HA present in natural bone structure includes semicrystalline structured carbonate with an approximate ratio of 5%–8% and is formed by nanodimensional particles. 

Matrices of cortical and trabecular parts of human bones are formed by two phases: Calcium HA and tricalcium phosphate (TCP:Ca3(PO4)2). Natural calcium HA which is the main phase forms a porous structure in cortical bones not less than a ratio of 50%. On the other hand, trabecular bones have a cage with porosity over 75%. Dimensions of the pores in human bones change between 100 and 500 μm and are filled with natural bone marrow. Bones are natural composites containing dentin, dental enamel, HA, protein, other organic materials, and water. Dental enamel is the hardest tissue containing highest amount of minerals among these tissues. Rigidity of bone tissue is more than the individual strengths of all its constituents. While the flexible structure of the collagen in bone structure prevents the brittle fracture of HA, HA prevents the entrance of collagen into plastic deformation area with its rigidity [13].

Surfacing hydroxyapatite on metallic materials is possible. By similar logic, to increase durability with low cost, characteristics of biomaterials can be improved with combinations such as hydroxyapatite PLA. Thus, we found that while these materials have usage limitations due to present disadvantages when used alone, it is possible to increase their efficiency and availability through various combinations.

HA-PLA filament produced biomaterials will be used for many branches in surgical department. For example; the implants produced for cranioplasty operations will be used for tabulas and orthopedics implants will be used for short and long bones in extremity executions. 

We acquired a success with our hydroxyapatite combination due to its compatibility with bone structure and machinability in reasonable melting point. We are hoping to find new hydroxyapatite combinations in service of medical use and present new models to be utilized by the researches of the biomedical sciences. 

In different studies in the literature, hydroxyapatite material is operated to be covered to Titanium and Ti-6AI-4V alloys with nano-coating techniques. Since this method needs high-level technology and high costs, we have modelled our study in different ways. However, we expect to get comparable results with these methods after continuing in vivo and in vitro studies. 

Our study called “Examining the Effects of 3D Produced Kits with Different Filament Materials over Rats” is a TÜBITAK 3501 project and approved by Kütahya Health Sciences University Animal Testing Ethics Committee. The purpose of the study is to research animal testings of the HA-PLA filament produced implants.
